# Stingray epidermal microbiomes are species-specific with local adaptations

**DOI:** 10.3389/fmicb.2023.1031711

**Published:** 2023-03-02

**Authors:** Emma N. Kerr, Bhavya Papudeshi, Miranda Haggerty, Natasha Wild, Asha Z. Goodman, Lais F. O. Lima, Ryan D. Hesse, Amber Skye, Vijini Mallawaarachchi, Shaili Johri, Sophia Parker, Elizabeth A. Dinsdale

**Affiliations:** ^1^Flinders Accelerator for Microbiome Exploration, College of Science and Engineering, Flinders University, Adelaide, SA, Australia; ^2^California Department of Fish and Wildlife, San Diego, CA, United States; ^3^Department of Biology, San Diego State University, San Diego, CA, United States; ^4^Hopkins Maine Station, Stanford University, Stanford, CA, United States

**Keywords:** stingray, elasmobranch, epidermis, microbiome, mucus, metagenomics

## Abstract

Marine host-associated microbiomes are affected by a combination of species-specific (e.g., host ancestry, genotype) and habitat-specific features (e.g., environmental physiochemistry and microbial biogeography). The stingray epidermis provides a gradient of characteristics from high dermal denticles coverage with low mucus to reduce dermal denticles and high levels of mucus. Here we investigate the effects of host phylogeny and habitat by comparing the epidermal microbiomes of *Myliobatis californica* (bat rays) with a mucus rich epidermis, and *Urobatis halleri* (round rays) with a mucus reduced epidermis from two locations, Los Angeles and San Diego, California (a 150 km distance). We found that host microbiomes are species-specific and distinct from the water column, however composition of *M. californica* microbiomes showed more variability between individuals compared to *U. halleri.* The variability in the microbiome of *M. californica* caused the microbial taxa to be similar across locations, while *U. halleri* microbiomes were distinct across locations. Despite taxonomic differences, Shannon diversity is the same across the two locations in *U. halleri* microbiomes suggesting the taxonomic composition are locally adapted, but diversity is maintained by the host. *Myliobatis californica* and *U. halleri* microbiomes maintain functional similarity across Los Angeles and San Diego and each ray showed several unique functional genes. *Myliobatis californica* has a greater relative abundance of RNA Polymerase III-like genes in the microbiome than *U. halleri*, suggesting specific adaptations to a heavy mucus environment. Construction of Metagenome Assembled Genomes (MAGs) identified novel microbial species within *Rhodobacteraceae*, *Moraxellaceae*, *Caulobacteraceae*, *Alcanivoracaceae* and Gammaproteobacteria. All MAGs had a high abundance of active RNA processing genes, heavy metal, and antibiotic resistant genes, suggesting the stingray mucus supports high microbial growth rates, which may drive high levels of competition within the microbiomes increasing the antimicrobial properties of the microbes.

## 1. Introduction

Host-associated microbiomes directly affect host health and development (Llewellyn et al., 2014; Apprill, 2017; Cullen et al., 2020; Malard et al., 2021). Microbiomes are host specific, vary with extrinsic factors, such as temperature and location and are impacted by climate change (Wilkins et al., 2019; Lima et al., 2020, 2022). Elasmobranchs, which includes sharks, rays, and skates, regulate the health of oceanic ecosystems (Sandin et al., 2008), but the connection between microbes and elasmobranch health is challenging to resolve. To date, elasmobranch-microbe relationships remain poorly understood, with the microbiomes of 37 shark and only 6 ray species out of 1,300 species being investigated (Perry et al., 2021).

*Batoidea* diverged from sharks between 200 and 229 million years ago, branching into modern rays about 140 million years ago (Aschliman et al., 2012). Batoidea includes over 600 species, about half of the diversity within Chondrichthyes (Aschliman et al., 2012; Kousteni et al., 2021). Twenty-two species of ray live along the coast of California accounting for over a quarter of elasmobranch diversity in the region (Ebert, 2003). In California, rays including *Myliobatis californica* (bat rays), *Urobatis halleri* (round rays), are meso-predators, feeding on small invertebrates, and serving as prey for larger elasmobranchs and marine mammals (Gray et al., 1997; Last et al., 2016). Rays disturb sediment to uncover prey, creating feeding pits which have a significant impact on benthic infauna communities by exposing otherwise sequestered resources and creating habitat for other organisms (van Blaricom, 1982). Sharks and rays regulate oceanic food webs and contribute to tourism economics (Newsome et al., 2004; Healy et al., 2020). Rays are key members of coastal ecosystems across the globe including sand flats, kelp forests, seagrass meadows, and coral reefs (Gray et al., 1997; O’Shea et al., 2012; Lyons et al., 2014). Elasmobranchs have long lifespans and late maturity which make them vulnerable to overexploitation, and many of these species are threatened globally (Domingues et al., 2018; Johri et al., 2019, 2020; Kousteni et al., 2021). Microbiome exploration in elasmobranchs using whole genome (shotgun) sequencing has allowed reconstruction of host genomes, which aids in resolving phylogenies, and can support conservation efforts (Doane et al., 2018; Johri et al., 2019). Elasmobranch microbiomes remain an important ecosystem of discovery, which has been focused on sharks while rays remain understudied (Kearns et al., 2017; Gonçalves e Silva et al., 2020; Pinnell et al., 2021; Clavere-Graciette et al., 2022).

The epidermis of Chondrichthyes is covered in dermal denticles, which are tooth-like placoid scales. Stingrays, unlike most sharks, have a thick layer of mucus with a reduced covering of dermal denticles. Both the dermal denticles and mucus act as the first defense against injury and invading pathogens (Meyer and Seegers, 2012), but where denticles are sparse, epidermal mucus serves as a barrier between the host and the environment. Proteases and antimicrobial peptides are present in ray mucus and reduce the survival of harmful microbes (Vennila et al., 2011). Stingray mucus, and the microbes within, produce antimicrobial molecules preventing infections of wounds resulting from feeding and mating (Kajiura et al., 2000; Conceição et al., 2012; Ritchie et al., 2017; Pogoreutz et al., 2019). The stingray epidermis, (and its unique mucus properties) serves as an interesting model system to compare with the microbiomes of sharks, which are covered in dermal denticles (Ritchie et al., 2017; Doane et al., 2022). Sharks with a dense denticle structure have microbiomes that are highly similar across individuals, species specificity, and show phylosymbiosis (Doane et al., 2017, 2020, 2022). The epidermis of teleost fishes is covered by a layer of thick mucus, similar to the mucus found on stingrays (Meyer and Seegers, 2012) and the microbiome of teleost fish is species-specific, but epidermal microbiomes generally have low similarity across individuals of the same species (Chiarello et al., 2018). Therefore, we predict that the mucus associated with rays will influence the taxonomy of the microbiome, but the functions of the ray microbiome will be similar to that of sharks, since they share similar metabolic characteristics, such as high levels of osmolytes (urea and TMAO (Trimethylamine N-oxide)) in the blood, and bioaccumulation of heavy metals (Withers et al., 1994a,b).

Stingray epidermal microbiomes vary between wild and captive individuals of the same species (Pinnell et al., 2021; Clavere-Graciette et al., 2022). The epidermal microbial community of cow-nose rays (*Rhinoptera bonasus*) from an aquarium, had lower diversity compared with of the surrounding environment suggesting the ray skin microbiome is selective (Kearns et al., 2017). *Hypanus americanus* (southern stingray) microbiomes are more similar to shark microbiomes than water column communities (Caballero et al., 2020). Yellow stingrays (*Urobatis jamaicensis*) microbiomes were distinctive across wild, aquarium-housed and aquarium-born rays. The wild caught rays had a lower abundance of Bacteroidetes, an abundant pelagic microbe, compared with those that were aquarium born, which suggests the filtered aquarium water environment has an impact on the skin microbiome (Pinnell et al., 2021). Leopard sharks (*Triakis semifasciata*) skin microbiomes did not show differences across captive and wild individuals, suggesting that skin properties could be contributing to microbiome stability (Goodman et al., 2022). There is evidence to suggest that captive status plays a major role in structuring elasmobranch mucus microbiomes, the driving force of these shifts in microbial communities is unknown. Therefore, we compare the effect of species-specific and habitat-specific drivers on the structure of the ray skin microbiomes in the wild.

We use whole genome (shotgun) sequencing metagenomics, which unlike 16S amplicon sequencing uses no primers and has allowed reconstruction of microbial genomes (Setubal, 2021). All ray microbiomes research has been conducting with amplicon sequencing (Kearns et al., 2017; Gonçalves e Silva et al., 2020; Pinnell et al., 2021; Clavere-Graciette et al., 2022), leaving novel microbes and functions of ray microbiomes understudied. Shotgun metagenomics requires higher sequence depth than 16S sequencing, but allows assemblely of the sequences together to construct Metagenomic Assembled Genomes (MAGs), which are near complete microbial genomes (Papudeshi et al., 2017; Tully et al., 2018). This process allows the identification of novel microbes that cannot be identified by 16S alone. Using reference independent assemblers avoids database bias, one of the limitations of shotgun metagenomics (Quince et al., 2017).

**Figure 1:**
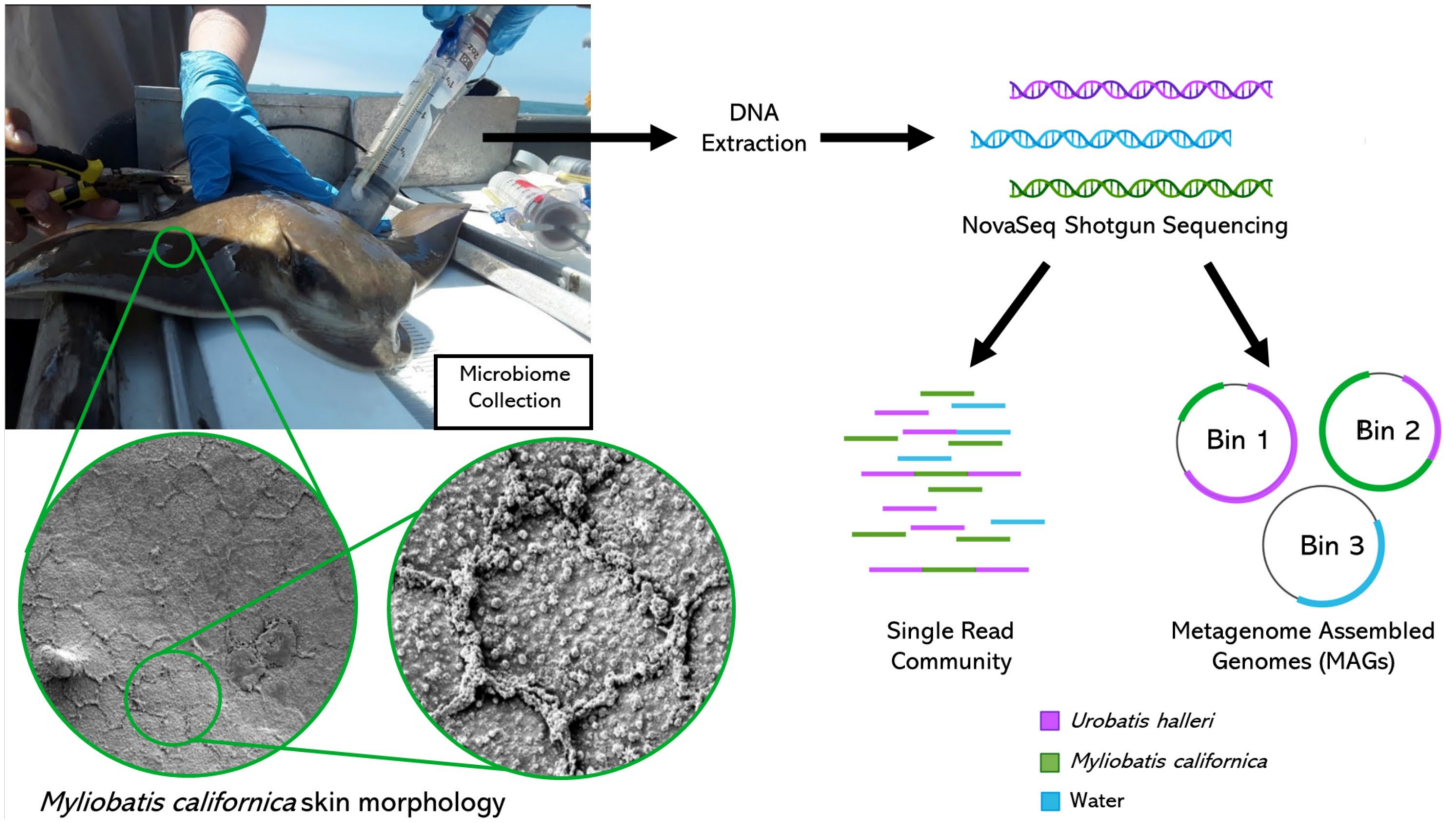
Metagenomics sample collection, processing, and bioinformatics analysis. Myliobatis californica skin scanning electron microscope images left: 1400X right: 6500X magnification.

We used shotgun metagenomics to describe the epidermal microbiome two species of wild Myliobatiforms, *Myliobatis californica*, and *Urobatis halleri* at two locations along the California coast. These rays have varying skin characteristics *Myliobatis californica* has reduced dermal denticles and high mucus production (Figure 1) and *Urobatis halleri* has less mucus and a higher covering of dermal denticles, which we hypothesize will be reflected in the characteristics in the skin microbiome. Our work contributes to filling the knowledge gaps on stingray microbiomes by identifying that stingray microbiome are species-specific. In *U. halleri*, location affected the microbiomes, but this effect was lost in the *M. californica* that had high mucus production.

## 2. Methods

Microbiome samples were collected from individuals along the California coast between Los Angeles Harbor and San Diego Bay. Sampling was conducted opportunistically during California Department of Fish and Wildlife halibut trawls in April and October of 2019. A small trawl was deployed for 10 min at a time at each site, with 5 deeper trawls (about 20 m) and 5 shallower (about 8 m). Elasmobranchs were retrieved and placed into containers with fresh seawater and sorted by species. Depending on location, zero to two elasmobranchs were collected per trawl, thus multiple trawls were required to obtain replicate microbial samples from each site and species. Microbiome samples were collected using a blunt ended two-way syringe (50 mL) called a “supersucker” (Figure 1). Four supersuckers, filled with sterile seawater which was flushed against the skin of the organism and microbial slurry was recollected back into the syringe, *via* a two-way valve collecting ~200 mL from each organism (Doane et al., 2017, 2020, 2022). The resulting microbial slurry is filtered through a 0.22 μm sterivex filter to capture microbes. All stingray microbiome samples were collected on the dorsal side of the organism avoiding the spine and providing consistency in sampling location. However, no difference has been observed between the dorsal and ventral sides of *R. bonasus* (Kearns et al., 2017). Water samples (about 2 liters per sample) were filtered through a 0.22 μm sterivex filter. Sterivex filters were stored on ice until they could be transported to a − 20°C freezer for long term storage. Sampling was conducted in compliance with IACUC guidelines (18–05-007D & 17–11-010D). Once samples were collected, all organisms were returned to the ocean. A total of 15 *M. californica* (Los Angeles n = 6 and San Diego *n* = 9) and 16 *U. halleri* (Los Angeles *n* = 8 and San Diego *n* = 8) had appropriate metagenomes for analysis.

DNA from host associated and water microbiomes were extracted using a modified column purification method with the Nucleospin tissue kit by Macherey-Nagel (Doane et al., 2017). Stingray metagenomes were sequenced at Microbial Genome Sequencing Center on the NovaSeq platform using the Illumina Nextera XT kit. Shotgun libraries of water samples were prepared using Swift 2S Plus Kit and manufacturer protocol (Swift Biosciences) sequenced at SDSU using the Illumina MiSeq platform. Metagenomics was used rather than meta-transcriptomics, as metagenomes describe the functional genes that are important for the microbiome (Dinsdale et al., 2008; Coelho et al., 2022) rather those that are being transcribed at the time of sampling. Sequences can be accessed using the BioProject number PRJNA837707; sample accession numbers range from SRR19392779 to SRR19392812 (Supplementary Table 1).

Metagenomes were annotated using a “snakemake” pipeline developed by Edwards (2020). Sequences were checked for quality using Prinseq software and reads with fewer than 60 base pairs, quality mean below 25 and more than 1 unidentified base were removed, and Poly A and T tails were trimmed by 5 base pairs (Schmieder et al., 2011). FOCUS and SUPERFOCUS was used to determine the taxonomic identity of the sequences and for the identification of functional genes (SEED Subsystem levels 1, 2 and 3) present in the metagenomes (Overbeek et al., 2014; Silva et al., 2014, 2016). To prevent unrelated microbes being grouped together as single “unknown family,” unknown reads were manually identified using the next highest positively identified classification (e.g., order, class, phylum). Read abundances were transformed into proportions to allow for analysis between metagenomes, which have variable number of sequences per library, which is preferred over rarefaction (Mc Murdie and Holmes, 2014; Quince et al., 2017; Calle, 2019) and data was transformed using a fourth root transformation (Lima et al., 2020). Unique diversity and Shannon diversity at the family level were compared across locations using Welch Two Sample t-test. Metagenomes were compared using a Bray–Curtis similarity matrix followed by PERMANOVA (Permutational multivariate analysis of variance) and PERMDISP (Permutational analysis of multivariate dispersions) (Anderson, 2017; Anderson et al., 2017) which were used to test for significant differences in microbiome across host species and locations. PERMANOVA takes a permutation approach to identify whether the microbial community is different across variables (host species or location) and SIMPER (similarity of percentages) identifies which microbial taxa or gene function was contributing the differences. PERMDISP calculates a centroid for the group of samples (i.e., all *U. halleri* metagenomes for example) and calculated the distance from the centroid to each of the samples within the group, the larger number the greater the variation between microbiomes. PCO (Principal Component Ordination) was used to visualize relationships between the microbiomes at family and SEED Level 3 Subsystems. ANOVA (analysis of variance) and Tukey–Kramer test were used to identify significant differences between functional gene potential of ray microbiomes and the water column (Lima et al., 2022). All multivariate statistical tests and diversity indices were conducted using Primer 7 (7.0.17) with PERMANOVA+ (Clarke and Gorley, 2015). All univariate statistics and visualizations were conducted with R using the ggplot2 package (Wickham et al., 2016). While there is constant renaming of microbial groups, for example, Proteobacteria recently being renamed Pseudomonadota phylum (Oren and Garrity, 2021), we have reported the taxonomy as it appears in NCBI.

Metagenome Assembled Genomes (MAGs) were co-assembled using all 34 metagenomes in this study. Reads (about 150 bp) are merged into longer sequences called contigs using Megahit (total 3,870,948 contigs assembled (Li D. et al., 2015). Contigs >1,500 base pairs (102,557 contigs) were binned into 36 bins with 95,155 contigs using Metabat2. Binning uses the characteristics of each contigs to group similar contigs into a ‘bin’. These characteristics include, contig coverage, GC content, Kmer frequencies (Papudeshi et al., 2017; Kang et al., 2019). GraphBin was used to refine binning by utilizing the assembly graph connections, increasing the number of contigs included in the 36 bins to 570,096 contigs (Mallawaarachchi et al., 2020). CheckM identified 16 of the refined bins to have >70% completeness using bacterial marker genes (Parks et al., 2015). Five bins contained <10% contamination (Parks et al., 2015, 2017; Papudeshi et al., 2017) and these bins are described to meet high quality bins using the minimum information for metagenomic assembled genomes (Bowers et al., 2017). The five high quality bins were uploaded to PATRIC (Pathosystems Resource Integration Center) where the relative abundance of SEED level 3 functional genes was transformed by squareroot, and a dendrogram heatmap was used to compared across bins (Aziz et al., 2008; Overbeek et al., 2014; Clarke and Gorley, 2015; Davis et al., 2020). The development of MAGs from single read sequences enables the annotation of entire genes and operons, thus providing improved gene descriptions and whereas metagenomes would be annotated with a metagenomic tool, MAGs are annotated with genomic tools, such as PATRIC. PATRIC’s similar genome finder identified the most similar reference genome, which may be a MAG, to which each bin was compared using FastANI (Jain et al., 2018; Davis et al., 2020).

A tissue sample was collected using a small 6 mm biopsy punch on the dorsal surface of a captive *M. californica* individual (not included in the microbial analysis in this study). The tissue samples were rapidly frozen in liquid nitrogen. They were removed frozen and then dropped into 2.5% glutaraldehyde in a 0.1 mol cacodylate buffer. They were then fixed overnight at room temperature, washed, and then dehydrated throughout a degraded series of alcohols. The samples were critical point dried with Sam Dri critical point dryer, mounted onto carbon-coated stubs, coated with 6 nm platinum, and observed in Quantas 450 FEG SEM. Three to five high-resolution images (100x magnification) were taken.

## 3. Results

### 3.1. Microbial taxonomic composition

Stingray microbiomes (*n* = 31) yielded 116,346,596 high quality sequences, with an average of 3,753,116 sequences per sample (Supplementary Table 1). *M. californica* (*n* = 15) and *U. halleri* (*n* = 16) microbiomes were composed of 415 microbial families. Water samples from Los Angeles (*n* = 1) and San Diego (*n* = 2) yielded 1,051,965 sequences with an average of 350,655 sequences per sample.

**Figure 2:**
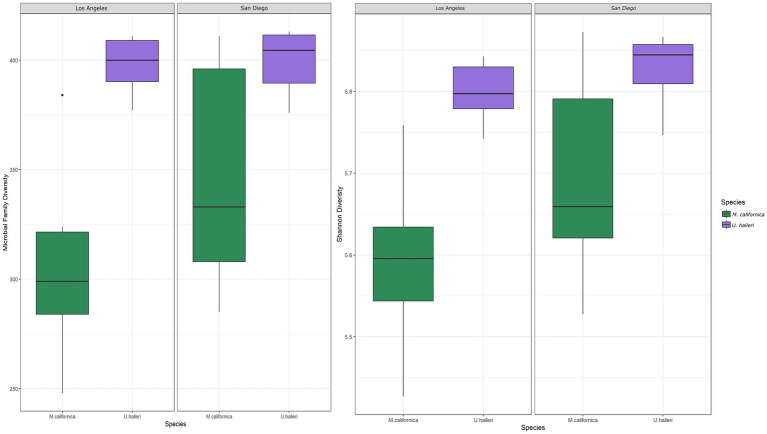
Boxplots depicting the differences in **(A)** total number of microbial families and **(B)** Shannon diversity between host species and location.

**Figure 3:**
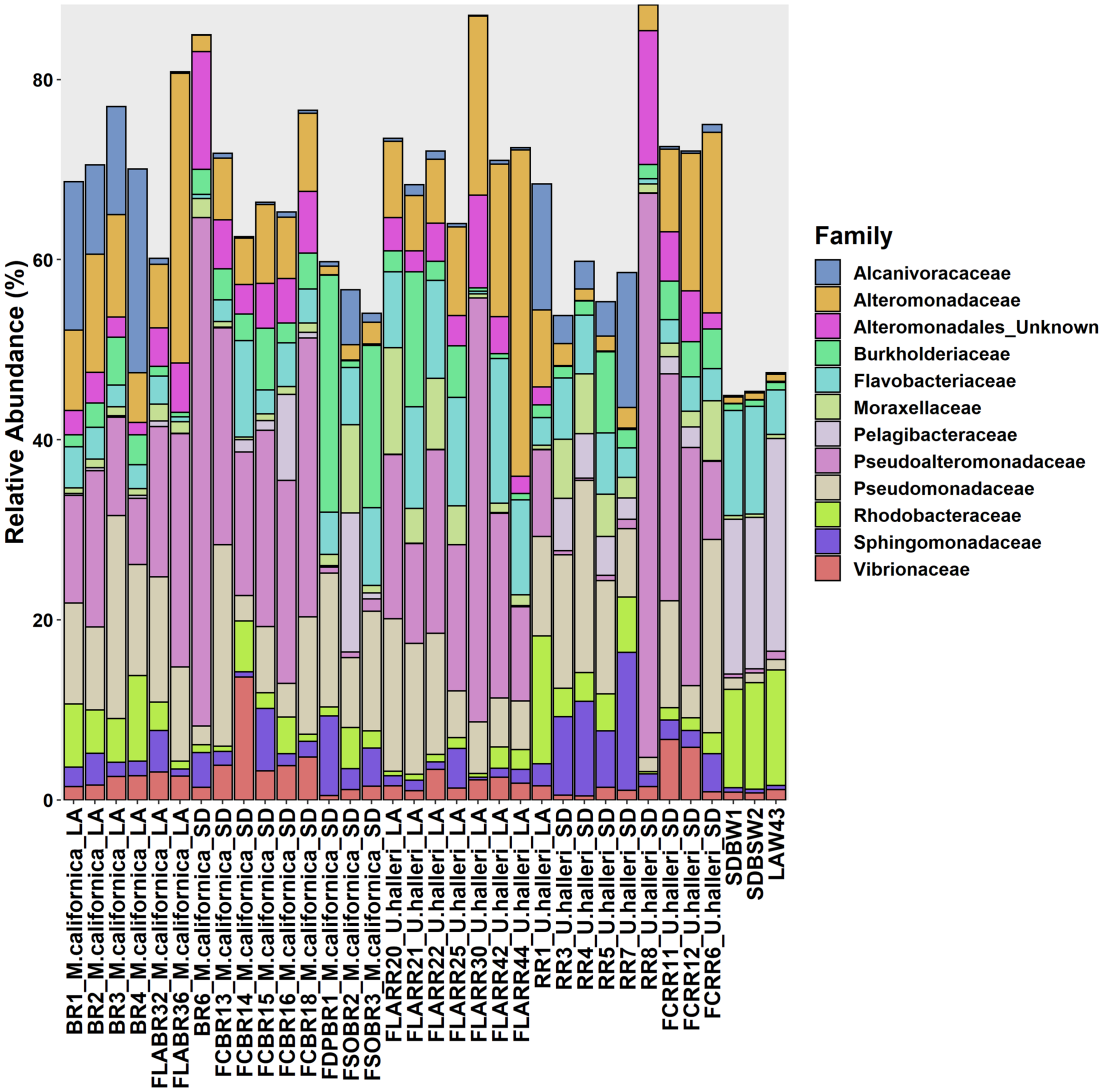
Variation of microbial families across *M. californica*, *U. halleri* and seawater microbiomes. Rare taxa are excluded from this graph, only microbes present with a relative abundance of 10% or greater in at least one sample are included. Samples appear in the same order as Supplementary Table 1.

*Myliobatis californica* microbiomes contained fewer unique families (mean 329.1 ± 48.8) and had lower Shannon diversity (H = 5.6 ± 0.13) than *U. halleri* microbiomes (mean families 398.44 ± 13.8 and H = 5.81 ± 0.04). *U. halleri* and *M. californica* had significantly different evenness (*t* = 3.80 *p* < 0.001)*. Myliobatis californica* and *U. halleri* microbiomes showed no significant difference in Shannon diversity between San Diego and Los Angeles locations (*t* = −1.737, df = 11.651, value of *p* = 0.108 and *t* = −1.602, df = 13.89, value of *p* = 0.1316 respectively) (Figure 2). Twelve families were present in host and water microbiomes with a relative abundance of ≥10% in at least one sample, but the relative abundance of these families varied between the rays and water column (Figure 3). Eleven of the most abundant families belong to Proteobacteria. Within Proteobacteria, six of the 12 most abundant microbes belong to the Gammaproteobacteria clade. *Alteromonadaceae*, *Pseudoalteromonadaceae*, *Pseudomonadaceae*, *Sphingomonadaceae*, and *Vibrionaceae* are present in greater relative abundance in host microbiomes compared with the water column. A novel *Alteromonadaceae* family was identified in greater relative abundance in ray microbiomes compared to the water column (Figure 3).

**Figure 4:**
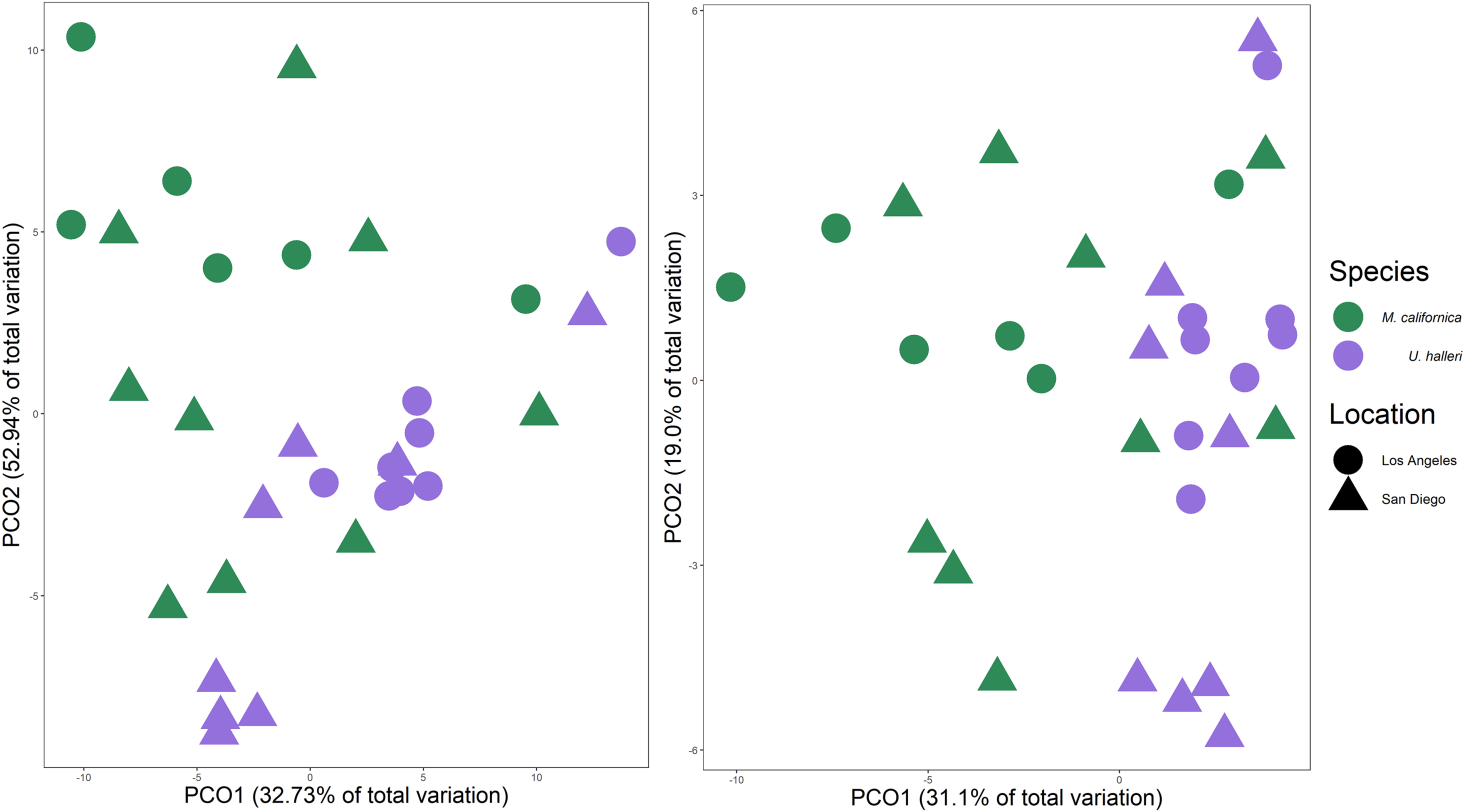
Principal coordinate analysis of Bray–Curtis similarity between **(A)** taxonomic composition and **(B)** functional gene potential (SEED Level 3 Subsystems) of *M. californica* and *U. halleri* microbiomes across sampling locations, showing the variation between the two species microbiome and the larger variation in microbiome that occurred across the individual *M. californica*.

*Myliobatis californica* and *U. halleri* microbial families were significantly different from each other and the water column, indicating species specificity (PERMANOVA *p* = 0.001, df = 2, Pseudo-*F* = 5.449). *M. californica* microbiomes were not significantly different between San Diego and Los Angeles (PERMANOVA *p* = 0.123, df = 1, Pseudo-*F* = 1.4083). In contrast, *U. halleri* microbiomes were significantly different between the two locations (PERMANOVA *p* = 0.019, df = 1, Pseudo-*F* = 3.8433) (Figure 4). *M. californica* and *U. halleri* microbiomes were 15.84% dissimilar to each other and *Pseudoalteromonadaceae* was the highest contributor to differences between hosts contributing 1.29% of the difference between microbiomes. *M. californica* microbiomes had greater variance than *U. halleri* microbiomes (PERMDISP *p* = 0.003, *F* = 19.58, df = 2) (Table 1; Figure 4), which was confirmed with a SIMPER analysis showed *M. californica* had an 82.67% taxonomic similarity between individuals, whereas *U. halleri* were 88.56% similar between individuals.

**Table 1:** Pairwise PERMDISP comparisons between host species from each location.

Microbial Family Pairwise Comparisons	*t*	*P perm*	SEED Level 3 Function Pairwise Comparisons	*t*	P perm
*M. californica* Los Angeles, *M. californica* San Diego	0.36	0.751	*M. californica* Los Angeles, *M. californica* San Diego	0.27	0.826
*M. californica* Los Angeles, *U. halleri* San Diego	1.88	0.166	*M. californica* Los Angeles, *U. halleri* San Diego	1.75	0.185
*M. californica* Los Angeles, *U. halleri* Los Angeles	3.63	0.012	*M. californica* Los Angeles, *U. halleri* Los Angeles	3.84	0.003
*M. californica* San Diego, *U. halleri* San Diego	2.96	0.035	*M. californica* San Diego, *U. halleri* San Diego	2.82	0.024
*M. californica* San Diego, *U. halleri* Los Angeles	5.61	0.002	*M. californica* San Diego, *U. halleri* Los Angeles	6.1	0.001
*U. halleri* San Diego, *U. halleri* Los Angeles	1.56	0.355	*U. halleri* Los Angeles, *U. halleri* San Diego	2.29	0.088

Highlighted comparisons are significant (*p* < 0.05).

### 3.2. Functional potential

**Figure 5:**
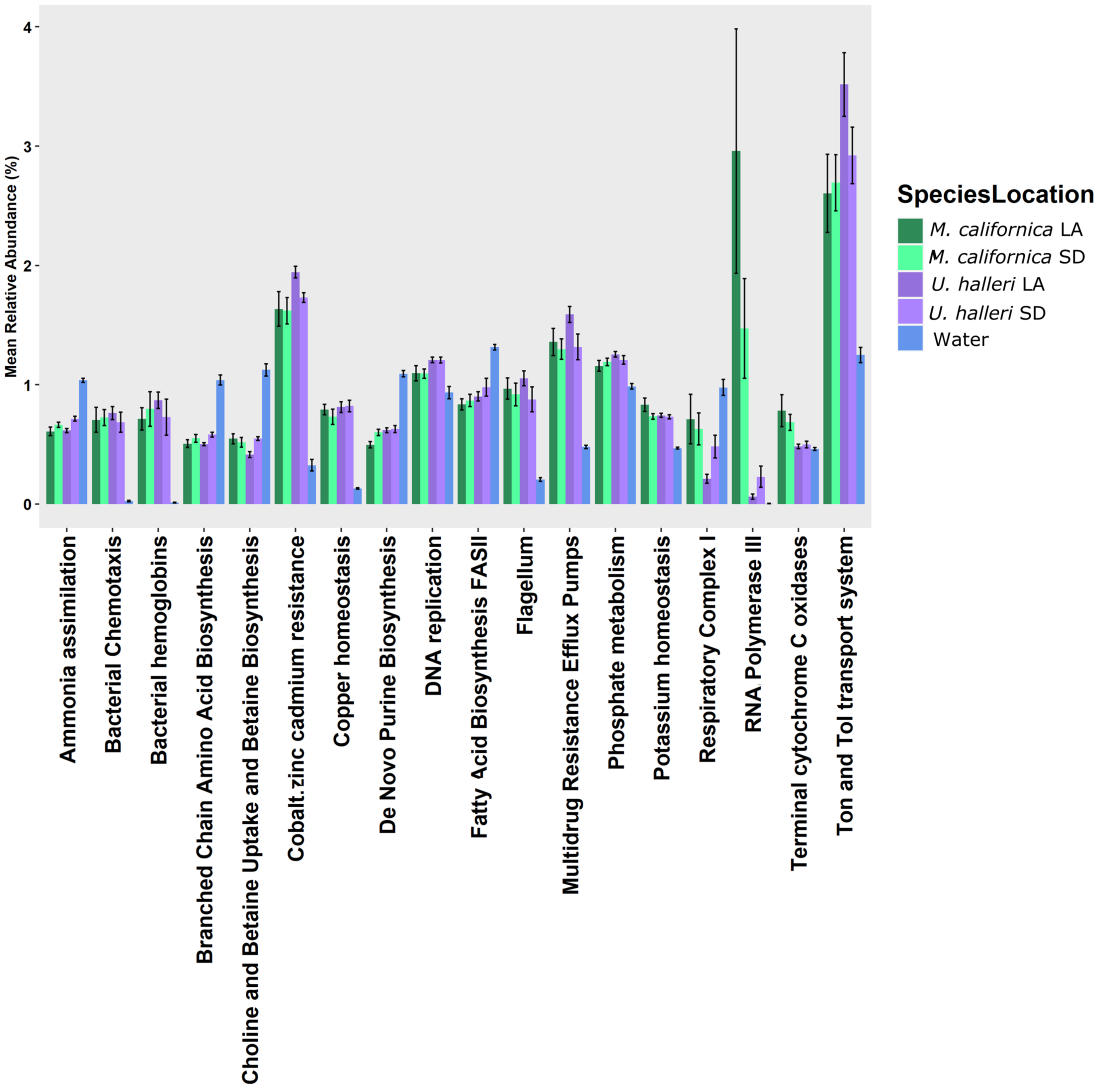
The relative abundance of functional genes (Level 3 SEED Subsystems) with >1% that showed a variation with the water column microbes.

The functional potential (SEED Level 3 Subsystems) of the skin microbiome was different between host species (PERMANOVA *p* = 0.001, df = 1, Pseudo-*F* = 5.0761). Neither ray species had significantly different SEED Level 3 functional potential between locations (PERMANOVA *p* = 0.121, df = 1, Pseudo-*F* = 1.4239 and *p* = 0.059 df = 1, Pseudo-*F* = 2.254 for *M. californica* and *U. halleri* respectively). SIMPER showed the functional potential of *M. californica* microbiomes were 89.56%, and *U. halleri* microbiomes were 93.16% similar. *M. californica* microbiome functions were 16.18% and *U. halleri* microbiomes were 15.15% dissimilar to the water column microbes. Host microbiome functional potential were 10.03% dissimilar. *Myliobatis californica* has significantly higher variance within the microbiome than *U. halleri* (PERMDISP *p* = 0.002, df = 3, *F* = 9.74). SIMPER analysis identified RNA Polymerase III-like genes accounting for the greatest difference (0.69%) between host microbiome functional potential. Out of 1,243 Level 3 functional genes, 18 have a relative abundance of ≥1% in at least one sample and vary across rays and water column. Bacterial chemotaxis, bacterial hemoglobin, cobalt-zinc-cadmium resistance, copper homeostasis, flagellum, multidrug resistance efflux pumps, RNA polymerase III-like, and Ton and Tol transport system genes are overrepresented in host microbiomes compared with the water column microbes (Figure 5). All high abundance gene pathways were significantly different between *M. californica* and the water column (ANOVA *p* < 0.05, Tukey–Kramer *p* < 0.05) except respiratory complex I (Tukey–Kramer *p* = 0.3) and terminal cytochrome C oxidase (Tukey–Kramer *p* = 0.06). *Urobatis halleri* microbiomes had significantly different pathways from the water column (ANOVA p < 0.05, Tukey–Kramer p < 0.05) except RNA Polymerase III-like and terminal cytochrome C (Tukey–Kramer *p* = 0.98 and *p* = 0.96 respectively) (Figure 5; Supplementary Table 2).

### 3.3. Metagenome assembled genomes

Across all stingray and water microbiomes, cross assembly of 34 metagenomes yielded five high-quality MAGs (Bins 9, 16, 17, 31, and 33) spanning a range of bacterial phyla. Bin 9 featured an 84.5% complete genome 2,998,016 bp in length from 679 contigs, with 3.14% contamination and 33.33% strain heterogeneity. Bin 16 featured a 74.14% complete genome 2,866,836 bp in length from 470 contigs with 0 contamination and 0 strain heterogeneity. Bin 17 featured an 86.13% complete genome 3,699,146 bp in length from 136 contigs with 0.84% contamination and 33.33% strain heterogeneity. Bin 31 featured a 93.4% complete genome 2,583,396 bp in length from 514 contigs with 3.27% contamination and 72.5% strain heterogeneity. Bin 33 featured an 89.94% complete genome 2,371,543 bp in length from 299 contigs with 4.69% contamination and 93.33% strain heterogeneity (Supplementary Table 3). Three bins (16, 17, 33) were > 95% similar to existing genomes, the remaining two (Bin 9 and 31) are novel species <95% similar to existing genomes (Supplementary Table 3).

**Figure 6:**
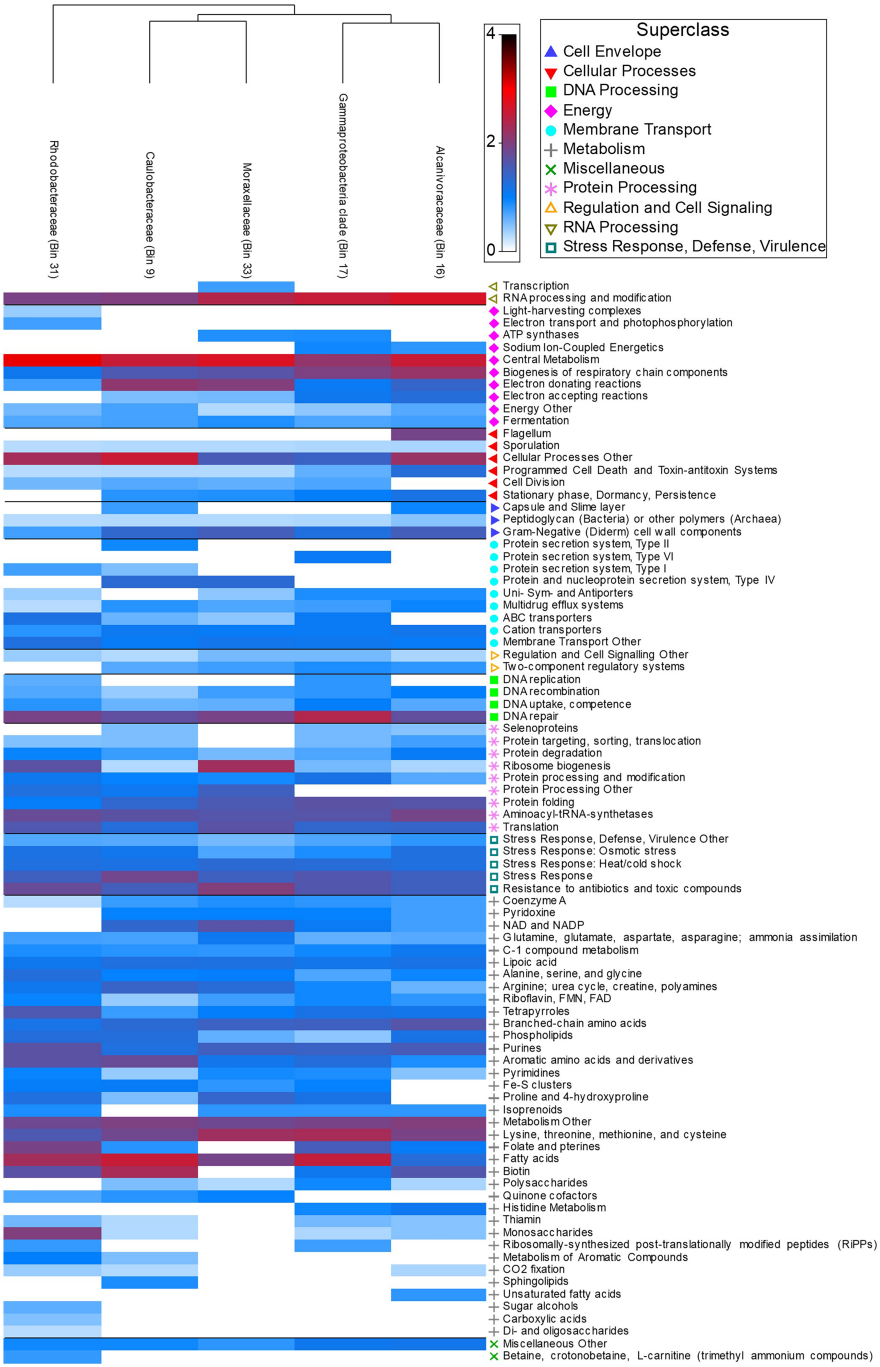
Heatmap depicting relative abundance of functional genes (Level 3 Subsystems) grouped into broader Level 1 Superclass present in host associated MAGs.

Functional pathways (SEED Subsystem: Level 1) featured a high proportion of active metabolism genes (39.03% ± 5.17) across all five MAGs. Utilization of monosaccharides (0.88% ± 1.74), di- and oligosaccharides (0.02% ± 0.03), and sugar alcohols (0.08% ± 0.17) occurred in low abundance across species and were most abundant in *Rhodobacteraceae* (Figure 6). In contrast, utilization of more complex polysaccharides was present in all species excluding *Rhodobacteraceae* (0.26% ± 0.34) (Figure 6). *Rhodobacteraceae* and *Moraxellaceae* also showed higher levels of ribosome biogenesis (Figure 6). DNA repair (3.83% ± 1.07), central metabolism (6.88% ± 1.74), and RNA processing and modification (5.44% ± 1.54) were among the most abundant active genes across species, with greater abundance in *Alcanivoracaceae*, Gammaproteobacteria, and *Rhodobacteraceae* bins (Figure 6). Furthermore, pathways involved in stress response, defense, and virulence were abundant (8.58% ± 0.36), including specific genes related to heat/cold shock (1.59% ± 0.13), osmotic stress (1.09% ± 0.47), resistance to antibiotic and toxic compounds (2.88% ± 0.78) and multidrug efflux systems (0.53% ± 0.30) were also active.

## 4. Discussion

We demonstrated that *Myliobatis californica* (bat ray) and *U. halleri* (round ray) microbiomes are species-specific, and distinct from the water column, regardless of sampling location showing that host phylogeny is an important selection pressure for the microbiome. Consistent with epidermal microbiomes of elasmobranchs including *Alopias vulpinus* (thresher sharks), *Triakis semifasciata* (leopard sharks), *Rhincodon typus* (whale sharks), and *Aetobatus narinari* (spotted eagle rays), which show a pattern of species specificity and host selection(Larsen et al., 2013; Doane et al., 2017, 2022; Storo et al., 2021). The microbes selected by *M. californica* and *U. halleri* were from the Proteobacteria phylum including *Pseudoalteromonadaceae, Alcanivoraceae*, *and Pseudomonadaceae* which is consistent with other ray species such as *Rhinoptera bonasus* (cownose ray), *Gymnura altavela* (butterfly ray) and *Dasyatis hypostigma* (groovebelly ray) (Kearns et al., 2017; Gonçalves e Silva et al., 2020). Cultured isolates from *R. bonasus* mucus include *Pseudoalteromonas* sp., *Alteromonas* sp., and *Vibrio* sp. which are recovered in our metagnomes (Ritchie et al., 2017). *Myliobatis californica* microbiomes have a significantly higher intra-species microbiome variance than *U. halleri*, suggesting fine-scale feature of the host epidermis and potential mucus turnover is affecting microbiome structure.

*Myliobatis californica* microbiomes are variable and show no significant difference in microbial taxonomy or functional potential across locations. *M. californica* are dispersed along the California coast and migrate up to 259 km during the summer to mate (Gong, 2022). Traveling large distances may obscure location specific effects on the microbiome. However, the microbial richness of the *M. californica* was lower compared with *U. halleri* suggesting selection *via* skin characteristics rather than migrative behavior. We suggest that the highly variable microbiome is associated with mucus production. We observed large amounts of mucus on *M. californica*, and while features of this mucus have not been measured specifically, mucus is consistently being produced and sloughed off as marine organisms propel through the water (Parrish and Kroen, 1988). *Manta birostris* (giant manta ray) and *T. semifasciata* (leopard shark) mucus has high isotopic turnover compared to other tissues, suggesting a highly variable environment for microbes (Malpica-Cruz et al., 2012; Burgess et al., 2018). Soluble fractions of sea bream (*Sparus aurata*) mucus had high carbon isotope turnover within 12 h of a diet switch, suggesting that soluble fractions of mucus are continuously produced and shed (Ordóñez-Grande et al., 2020). High turnover of mucus serves as a selective pressure, and only microbes that are adapted to replicate quickly would be able to survive causing the microbiome to have lower diversity and higher intraspecies variation (Figures 2, 4).

The microbiome characteristics of *U. halleri* suggests the mucus and epidermis condition are different to *M. californica*. The *U. halleri* microbiomes were consistent across individuals and were location specific. *U. halleri* migrate shorter distances (about 30 km) and have small home ranges but maintain high gene flow across southern California (Plank et al., 2010). Thus, low gene flow and genetic drift between the host from different locations that then modifies the microbiome is not likely to be the cause of the differences in microbiomes (Nemergut et al., 2013). Similar to *U. halleri*, the microbial taxonomic composition in *Carcharhinus melanopterus* (black tip reef sharks) was location specific across five reef sites (Pogoreutz et al., 2019). *C. melanopterus* also have small home ranges compared to pelagic shark species, and transfer microbes between individuals during feeding and mating (Mull et al., 2010; Pogoreutz et al., 2019). Location specific diet affected the gut microbiomes of detritivores feeding fish (Wu et al., 2012) and could be a feature of the *U. halleri* microbiomes. Divergent selection pressures between locations, such as interactions with the water column microbes, which show biogeography (Haggerty and Dinsdale, 2017) or physicochemical variables could play a role. The skin-microbiomes of three fish species in the Amazon identified high degree of co-correlations between skin and water column microbes, but very few co-correlations between the skin microbes and physiochemical variables suggesting that the host is filtering a sub-selection of the microbes in the surrounding (Sylvain et al., 2020). Despite significantly different microbial taxonomic abundances, microbial family diversity and Shannon diversity were maintained at both locations. Therefore, we suggest that turnover rate of the mucus of *U. halleri* is lower than *M. californica* and the skin microbiomes may be interacting with the water-column microbes, similar to teleost fish.

The epidermis and mucus production of sharks, rays, and teleost fish is variable and affects the microbiome. Sharks have minimal mucus and dense coverage of denticles (Meyer and Seegers, 2012), which leads to a highly structured microbiome (Doane et al., 2017, 2022). Teleost fish have epidermal scales which are covered with mucus which is reflect in microbiomes that has high alpha diversity high variability between individuals of the same species (Chiarello et al., 2015, 2018). Proteases in stingray mucus have antibacterial and antifungal activities, but the effects of stingray antimicrobial proteins on microbiome composition have not been explored (Vennila et al., 2011). In fish, the skin mucus microbes are species- specific in nature, but interaction networks showed high connectivity between the fish and water column microbes, suggesting they are affected by the microbes and environmental features of the location (Sylvain et al., 2020). Elasmobranchs demonstrate phylosymbiosis in their epidermal microbiomes, but signals are weak or absent in mucus microbiomes of fish, suggesting that mucus is more influenced by environment than denticle covered surfaces (Chiarello et al., 2018; Pollock et al., 2018; Doane et al., 2020).

Neither *M. californica* nor *U. halleri* had significantly different functional potential between locations, suggesting functionally redundancy (Louca et al., 2018). Functional redundancy describes that metabolic functions can be carried out by taxonomically distinct microbes (Louca et al., 2018). *Trakiatis semifasciata* (leopard shark) microbiomes maintain functional redundancy throughout time, even with taxonomic fluctuations (Doane et al., 2022). Functional genes present in high abundance on the stingrays, such as heavy metal resistant genes, Ton and Tol transporters, relative to the water column are consistent with other shark metagenomes (Doane et al., 2017, 2020). *Trakiatis semifasciata* and *A. vulpinus* (thresher sharks) both had higher relative abundance of cobalt zinc and cadmium resistance, and Ton and Tol transport system genes compared with the surrounding water column (Doane et al., 2017, 2022).

An RNA Polymerase III- like gene was highly abundant in *M. californica* microbiomes compared to both *U. halleri* and the water column. Our bioinformatic pipeline compares the stingray metagenomes to Chondrichthyan host genomes (< 10% of reads removed before microbial annotation), thus removing the possibility that host contamination was contributing to the presence of the high relative abundance of RNA Polymerase III-like genes in the metagenomes. The RNA Polymerase III-like gene is a eukaryotic specific gene but shows similarity with other RNA Polymerase subunits (i.e., I and II) in prokaryotes and viruses (Allison et al., 1965; Sweetser et al., 1987) and thus we suggest the RNA- Polymerase III-like gene is of prokaryote origin, but divergent Polymerase genes that are currently represented in the database. This is consistent with the high novelty that was identified in the MAGs that we constructed from the stingray metagenomes. In *Saccharomyces*, RNA Polymerase III is active in the presence of abundant nutrients, leading to rapid growth, whereas in nutrient depleted environments RNA Polymerase III activity declines (Roberts et al., 2003). Growth rate due to genetic variation is not well understood but has been correlated with high copy numbers of ribosomal RNA operons (*rrn*) (Ciara et al., 1995; Klappenbach et al., 2000). High *rrn* copy numbers in a bacterial isolate from high nutrient marine environment suggests a link between RNA genes and adaptations to high nutrient conditions (Lauro et al., 2009). Therefore, we suggest in a nutrient rich mucus layer of the stingrays, microbes are growing rapidly, which is reflected in a high relative abundance of RNA Polymerase genes. These genes constituted <1% of the genes in microbiomes from *T. semifasciata*, *R. typus, A. vulpinus,* and *Carcharodon carcharias* (great white shark) (Doane et al., 2017; Goodman et al., 2022; Pratte et al., 2022). Shotgun microbiome studies of teleost fish are currently limited to the gut (Legrand et al., 2020), thus making comparison with fish epidermal microbiome not possible. High relative abundance of RNA processing genes only in elasmobranchs with mucus and the high proportional abundance in the *M. californica* microbiome suggests mucus production and turnover are important structural feature of skin microbiomes and warrant future investigation.

Stingray MAGs had high completeness and low contamination but could only be annotated to the family level, highlighting novel bacterial species. The construction of MAGs identified *Moraxellaceae* and *Rhodobacteraceae*, both of which have been observed in captive *Rhinoptera bonasus* (cow-nose rays) (Kearns et al., 2017). Functional gene pathways including RNA processing, metabolism, and antimicrobial pathways were abundant in stingray MAGs. The RNA processing genes present in the MAGs were described as “active” by the PATRIC database algorithms and were highly similar to RNA genes in the NCBI database, supporting the single read data. The thick mucus layer on the batoids epidermis provide a high nutrient matrix for microbial growth (Shoemaker and LaFrentz, 2015) and while mucus properties were not measured, our data suggests variation in mucus turnover rate between the two stingray hosts. Antimicrobial genes present in MAGs signify interspecies competition within the stingray microbiomes consistent with competitive interaction of the microbes cultured from stingray mucus (Kearns et al., 2017; Ritchie et al., 2017; Gonçalves e Silva et al., 2020). The batoid mucus shows antibacterial action against human pathogens and expedites the healing processes of host wounds (Ritchie et al., 2017; Perry et al., 2021). The ubiquitous presence of antimicrobial genes across the MAGs raises the question of whether the antibiotic properties of the stingray mucus is being produced by the host or the microbial community. Multidrug resistance efflux pumps within the microbial genome provides resistance to antimicrobials (Piddock, 2006; Vila and Martinez, 2008; Li X.-Z. et al., 2015; Jang, 2016) and these are common in other elasmobranch microbiomes (Doane et al., 2017, 2022). A high abundance of antimicrobial resistance genes reflects the elevated abundance of antibiotics and toxic compounds within the mucus and the interspecific competition within the microbial community.

The microbiome of *M. californica* maintains taxonomic and functional stability across southern California. *Urobatis halleri* maintain functional gene potential but have significantly different taxonomy across locations despite high similarity between individuals. While the microbiome of the rays shared many characteristics with other elasmobranch species, the variation in β-diversity across ray species suggest variation in mucus turnover rates may be an important structuring feature of epidermal microbiomes and requires further investigation. Host microbiomes enriched in heavy metal resistance genes appears to be a signature of elasmobranch microbiome and may suggest changes in host health. The high levels of RNA Polymerase pathways, a signature of rapid microbial replication, combined with the high levels of antimicrobial resistance suggests stingray mucus promotes microbial competition.

## Data availability statement

The datasets presented in this study can be found in online repositories. The names of the repository/repositories and accession number(s) can be found in the article/Supplementary material.

## Ethics statement

The animal study was reviewed and approved by SDSU IACUC 18–05-007D & 17–11-010D.

## Author contributions

EK collected the samples, extracted the DNA and sequenced the metagenomes and conducted the analysis, and wrote the paper. EK and ED conceived the experiment. ED contributed to writing the manuscript. BP and VM constructed the MAGs and help with the bioinformatics. AS and NW helped with MAG analysis and visualization. MH facilitated the collection of the stingrays in association with California Fish and Wildlife. MH, SP, AG, RH, and SJ helped with the collection. LL helped with sequencing. All authors contributed to the article and approved the submitted version.

## Funding

We wish to thank the Stephanie Lo and Ben Billings Global Shark Research and Conservation fund for their support of the research. We acknowledge the funding from California Association of Ocean Affairs Science and Technology award to EK.

## Conflict of interest

The authors declare that the research was conducted in the absence of any commercial or financial relationships that could be construed as a potential conflict of interest.

## Publisher’s note

All claims expressed in this article are solely those of the authors and do not necessarily represent those of their affiliated organizations, or those of the publisher, the editors and the reviewers. Any product that may be evaluated in this article, or claim that may be made by its manufacturer, is not guaranteed or endorsed by the publisher.
