# Supplementary material for: Stingray epidermal microbiomes are species-specific with local adaptations
Source: Front Microbiol. 2023 Mar 2;14:1031711. doi: 10.3389/fmicb.2023.1031711 (PMC10017458; doi:10.3389/fmicb.2023.1031711)
Supplement: Supplementary file 1 [file Table_1.DOCX]

Supplementary Table 1: Sequencing characteristics for all metagenomes.

| Sample | Number of Sequences | Total Length (base pairs) | SRA Accession Number |
| --- | --- | --- | --- |
| BR1_*M. californica*_LA | 2,836,348 | 417,583,808 | SRR19392812 |
| BR2_*M. californica*_LA | 3,087,737 | 412,648,462 | SRR19392811 |
| BR3_*M. californica*_LA | 3,222,884 | 429,435,652 | SRR19392800 |
| BR4_*M. californica*_LA | 2,366,977 | 348,943,221 | SRR19392789 |
| FLABR32_*M. californica*_LA | 3,270,999 | 481,363,353 | SRR19392784 |
| FLABR36_*M. californica*_LA | 4,455,194 | 585,640,485 | SRR19392783 |
| BR6_*M. californica*_SD | 3,874,957 | 527,003,205 | SRR19392782 |
| FCBR13_*M. californica*_SD | 3,039,185 | 404,934,699 | SRR19392781 |
| FCBR14_*M. californica*_SD | 4,302,024 | 543,925,651 | SRR19392780 |
| FCBR15_*M. californica*_SD | 2,641,366 | 389,418,541 | SRR19392779 |
| FCBR16_*M. californica*_SD | 4,970,087 | 626,033,720 | SRR19392810 |
| FCBR18_*M. californica*_SD | 3,767,721 | 486,296,771 | SRR19392809 |
| FDPBR1_*M. californica*_SD | 2,645,824 | 350,666,845 | SRR19392808 |
| FSOBR2_*M. californica*_SD | 3,923,330 | 505,311,011 | SRR19392807 |
| FSOBR3_*M. californica*_SD | 3,158,635 | 421,845,873 | SRR19392806 |
| FLARR20_*U. halleri*_LA | 3,892,074 | 503,682,608 | SRR19392805 |
| FLARR21_*U. halleri*_LA | 3,262,778 | 419,680,365 | SRR19392804 |
| FLARR22_*U. halleri*_LA | 3,184,239 | 418,069,390 | SRR19392803 |
| FLARR25_*U. halleri*_LA | 4,672,533 | 580,369,873 | SRR19392802 |
| FLARR30_*U. halleri*_LA | 4,133,388 | 513,704,828 | SRR19392801 |
| FLARR42_*U. halleri*_LA | 3,957,020 | 542,437,760 | SRR19392799 |
| FLARR44_*U. halleri*_LA | 3,673,869 | 503,817,610 | SRR19392798 |
| RR1_*U. halleri*_LA | 5,499,533 | 749,123,698 | SRR19392797 |
| RR3_*U. halleri*_SD | 3,598,286 | 449,710,240 | SRR19392796 |
| RR4_*U. halleri*_SD | 5,204,519 | 707,235,278 | SRR19392795 |
| RR5_*U. halleri*_SD | 4,233,567 | 592,387,351 | SRR19392794 |
| RR7_*U. halleri*_SD | 4,188,561 | 581,233,486 | SRR19392793 |
| RR8_*U. halleri*_SD | 4,533,582 | 605,911,450 | SRR19392792 |
| FCRR11_*U. halleri*_SD | 2,667,563 | 352,045,492 | SRR19392791 |
| FCRR12_*U. halleri*_SD | 3,625,315 | 470,974,465 | SRR19392790 |
| FCRR6*_U. halleri_*SD | 4,456,501 | 614,810,049 | SRR19392788 |
| LAW43 water | 481,196 | 131,375,559 | SRR19392785 |
| SDBW1 water | 76,839 | 20,277,138 | SRR19392787 |
| SDBSW2 water | 493,930 | 139,108,939 | SRR19392786 |
