# Supplementary material for: Stingray epidermal microbiomes are species-specific with local adaptations
Source: Front Microbiol. 2023 Mar 2;14:1031711. doi: 10.3389/fmicb.2023.1031711 (PMC10017458; doi:10.3389/fmicb.2023.1031711)
Supplement: Supplementary file 2 [file Table_2.DOCX]

Supplementary Table 2: ANOVA and Tukey-Kramer results for Level 3 SEED Subsystem comparisons between host microbiomes and the water column.

| Gene | ANOVA p-value | Multiple R-squared | *M. californica* Tukey-Kramer p-value | *U. halleri* Tukey-Kramer p-value |
| --- | --- | --- | --- | --- |
| Ammonia assimilation | < 0.001 | 0.71 | < 0.001 | < 0.001 |
| Bacterial Chemotaxis | < 0.001 | 0.51 | 0.003 | 0.001 |
| Bacterial hemoglobins | 0 | 0.32 | < 0.001 | < 0.001 |
| Branched-Chain Amino Acid Biosynthesis | < 0.001 | 0.78 | < 0.001 | < 0.001 |
| Choline and Betaine Uptake and Betaine Biosynthesis | < 0.001 | 0.77 | < 0.001 | < 0.001 |
| Cobalt-zinc-cadmium resistance | < 0.001 | 0.75 | < 0.001 | < 0.001 |
| Copper homeostasis | < 0.001 | 0.66 | < 0.001 | < 0.001 |
| De Novo Purine Biosynthesis | < 0.001 | 0.79 | < 0.001 | < 0.001 |
| DNA-replication | < 0.001 | 0.42 | 0.04 | < 0.001 |
| Fatty Acid Biosynthesis FASII | < 0.001 | 0.43 | < 0.001 | 0.001 |
| Flagellum | < 0.001 | 0.45 | < 0.001 | < 0.001 |
| Multidrug Resistance Efflux Pumps | < 0.001 | 0.53 | < 0.001 | < 0.001 |
| Phosphate metabolism | < 0.001 | 0.37 | 0.006 | < 0.001 |
| Potassium homeostasis | < 0.001 | 0.54 | < 0.001 | < 0.001 |
| Respiratory Complex I | < 0.001 | 0.29 | 0.3 | 0.01 |
| RNA Polymerase III | < 0.001 | 0.37 | 0.04 | 0.98 |
| Terminal cytochrome C oxidases | 0 | 0.33 | 0.06 | 0.96 |
| Ton and Tol transport system | < 0.001 | 0.39 | 0.01 | < 0.001 |
