# Supplementary material for: Stingray epidermal microbiomes are species-specific with local adaptations
Source: Front Microbiol. 2023 Mar 2;14:1031711. doi: 10.3389/fmicb.2023.1031711 (PMC10017458; doi:10.3389/fmicb.2023.1031711)
Supplement: Supplementary file 3 [file Table_3.DOCX]

Supplementary Table 3: Average nucleotide identity between each stingray MAG and most similar genome identified by PATRIC’s Similar Genome Finder.

| Reference Genome | Query | GenBank Accession Number | Percent Identity |
| --- | --- | --- | --- |
| *Caulobacteraceae* | Bin_9 | DEWY01000001.1 | 80.7945 |
| Alcanivorax | Bin_16 | DFMU01000001.1 | 97.2609 |
| *Rheinheimera aquimaris* | Bin_17 | PNRD01000001.1 | 98.384 |
| *Rhodobacteraceae* | Bin_31 | JAMWZG010000001.1 | 77.9628 |
| Enhydrobacter | Bin_33 | LR733345.1 | 96.2021 |
