# Supplementary figures and images for: Stingray epidermal microbiomes are species-specific with local adaptations
Source: Front Microbiol. 2023 Mar 2;14:1031711. doi: 10.3389/fmicb.2023.1031711 (PMC10017458; doi:10.3389/fmicb.2023.1031711)

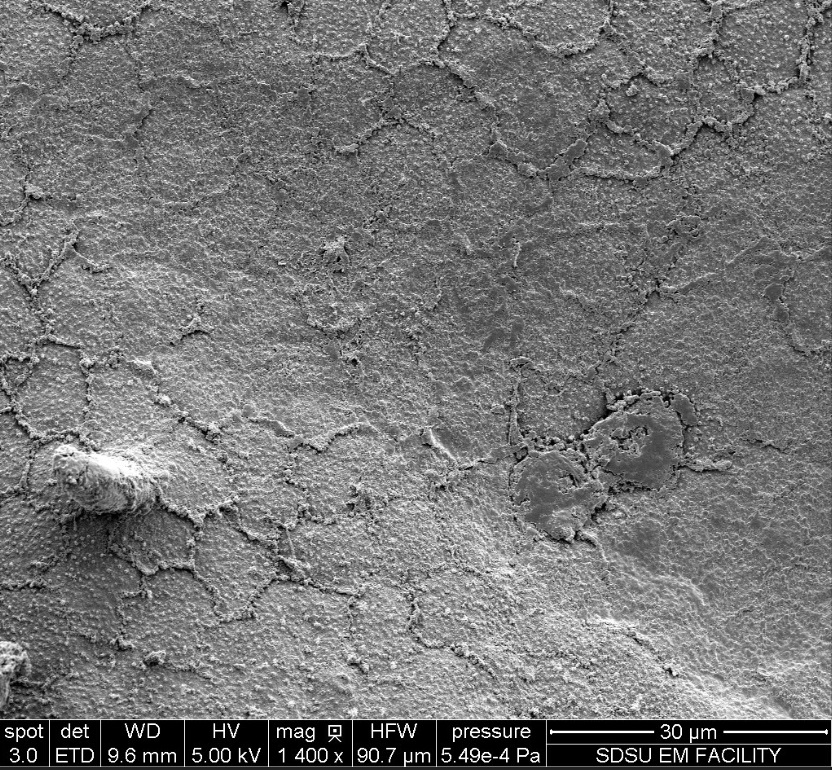

Supplement: Supplementary file 4 [file Figure_1.JPEG]
